# microRNA-451a promoter methylation regulated by DNMT3B expedites bladder cancer development via the EPHA2/PI3K/AKT axis

**DOI:** 10.1186/s12885-020-07523-8

**Published:** 2020-10-21

**Authors:** Beibei Liu, Wei Sun, Wuyue Gao, Liqiang Li, Zhenxue Cao, Xiaohuai Yang, Jianmin Liu, Yuanyuan Guo

**Affiliations:** Department of Urology, The First Affiliated Hospital of Bengbu Medical College, No. 287, Changhuai Road, Bengbu, 233000 Anhui People’s Republic of China

**Keywords:** Bladder cancer, DNMT3B, microRNA-451a, EPHA2, The PI3K/AKT signaling

## Abstract

**Background:**

The downregulation of microRNA (miR)-451a has been reported in bladder cancer (BCa) tissues. Herein, we elucidated the role of miR-451a in BCa with the involvement of DNA methyltransferase 3B (DNMT3B).

**Methods:**

We first screened the differentially expressed miRNAs from the serum of 12 BCa patients and 10 healthy controls in the BCa database GSE113486. Subsequently, we detected miR-451a expression and CpG island methylation of the promoter in BCa cells T24 and 5637 with DNMT3B knockdown. The downstream mRNAs of miR-451a were predicted by bioinformatics and KEGG enrichment analysis. Afterwards, the expression patterns of DNMT3B, miR-451a and erythropoietin-producing hepatocellular receptor tyrosine kinase class A2 (EPHA2) were altered in BCa cells to test the ability of cell proliferation, apoptosis, migration as well as invasion. Finally, the effect of miR-451a and DNMT3B was evaluated in vivo.

**Results:**

miR-451a was significantly reduced in serum of BCa patients and cell lines. Moreover, the expression of DNMT3B in BCa cells was significantly increased, thus promoting methylation of the miR-451a promoter, resulting in miR-451a inhibition. Additionally, we found that miR-451a targeted and negatively regulated EPHA2, while EPHA2 could activate the PI3K/AKT signaling, driving BCa cell growth and metastasis.

**Conclusions:**

Our study proposed and demonstrated that miR-451a downregulation mediated by DNMT3B is critical for proliferation, migration, and invasion of BCa, which may be beneficial for developing more effective therapies against BCa.

**Supplementary information:**

**Supplementary information** accompanies this paper at 10.1186/s12885-020-07523-8.

## Background

Bladder cancer (BCa), the most common and lethal cancer in the urinary system, holds accountable for estimated 81,400 new cases and 17,980 death in the USA in 2020 [1]. BCa is divided into two main categories, muscle-invasive BCa (accounting for 25%) and non-muscle invasive BCa (accounting for 75%), and the former one is treated by radical cystectomy, adjuvant chemotherapy or chemoradiotherapy, while the latter one is usually treated by transurethral resection and intravesical therapies [2]. About half of non-muscle invasive BCa are low grade, whereas most muscle-invasive or metastatic BCa are high grade [3]. To significantly halt the course of the malignancy, improvements should be achieved in the diagnosis and treatment of metastatic BCa.

MicroRNAs (miRNAs) are endogenous ~ 23 nt RNA transcripts that play vital gene-regulatory roles in animals and plants by matching mRNAs to direct their posttranscriptional repression [4]. Several miRNAs, including miR-205, miR-210, miR-124 and miR-214 have been elucidated as active players in BCa cell apoptosis, proliferation and differentiation [5]. Deep-sequencing-based BCa signatures conducted by Matsushita et al. suggested that clustered miRNAs, including miR-451a were downregulated in BCa tissues [6], with their specific role in BCa undetermined. Even though the mechanisms behind miRNA dysregulation in malignancies have not been clearly established, the silencing of several miRNAs has been suggested to correlated with epigenetic mechanisms, such as histone modification and DNA methylation [7]. For instance, it has been previously reported that upregulated heterochromatin protein 1γ in prostate cancer cells repressed the expression pattern of miR-451a by promoting H3K9 methylation at the promoter region of miR-451a [8]. Therefore, we hypothesized that the declines in miR-451a expression in BCa was also in a correlation with methylation of its promoter region. Interestingly, methylation of cytosine and guanine (CpG) islands contributed to the knockdown of miR-502-5p in BCa, while DNA methyltransferase 3B (DNMT3B) and miR-502-5p established a feedback loop in the modulation of BCa [9]. Also, the level of DNMT3B was significantly promoted in BCa tissues and cells relative to the normal controls, and DNMT3B siRNAs reduced BCa cell proliferation, migration and invasion, whereas stimulated cell apoptosis [10]. Thus, we could reasonably postulate that miR-451a plays a tumor suppressor role in BCa in a DNMT3B-dependent manner. Therefore, this study was undertaken to probe whether miR-451a regulates BCa growth through DNMT3B-mediated methylation and the downstream biomolecules. This study may provide new and promising therapeutic targets for BCa treatment.

## Methods

### Cell lines, antibodies, vectors and reagents

5-aza-dC purchased from Sigma-Aldrich Chemical Company (St Louis, MO, USA) was dissolved in DMSO and configured into 5 μM solution for further use. Urinary bladder transitional cell carcinoma cell T24 (#SCSP-536), urinary bladder grade II carcinoma cell 5637 (#TCHu 1), urinary bladder grade IV transitional cell carcinoma cell TCCSUP (#SCSP-571) and urinary bladder grade IV transitional cell carcinoma cell UM-UC3 (#SCSP-684, all BCa cell lines) were from Cell Bank of Shanghai Institute of Cells (Shanghai, China). Immortalized human uroepithelial cells SV-HUC-1 were from ATCC (Manassas, VA, USA, #CRL-9520). The antibodies used in the experiments were DNMT3B (#72335, Cell Signaling Technologies (CST), Beverly, MA, USA), phos-PI3K (#ab182651, Abcam, Cambridge, UK), phos-AKT1 (#44-621G, Thermo Fisher Scientific Inc., Waltham, MA, USA), EPHA2 (#37–4400, Thermo Fisher), KI67 (#9027, CST), glyceraldehyde-3-phosphate dehydrogenase (GAPDH, #ab181602, Abcam), horseradish peroxidase (HRP)-labeled goat anti-mouse (#G-21040, Thermo Fisher) or goat anti-rabbit IgG (#G-21234, Thermo Fisher). Terminal deoxynucleotidyl transferase (TdT)-mediated dUTPbiotin nick end labeling (TUNEL) kit was purchased from Promega (#G3250, Madison, WI, USA). 5-Ethynyl-2′-deoxyuridine (EdU) cell proliferation detection kit was obtained from Solarbio (#CA1170, Beijing, China). DMEM and FBS were from Gibco (Carlsbad, CA, USA). Short hairpin RNAs (shRNAs) targeting DNMT3B and corresponding Scramble shRNA (sh-NC), miR-451a inhibitor, inhibitor NC, miR-451a mimic, mimic NC (Mock), oe-NC and oe-EPHA2 were synthesized and purchased from OriGene Technologies (Beijing, China). Primer sequences used for quantitative reverse transcriptase-PCR (RT-qPCR) were synthesized by Shanghai Sangon Biological Engineering Technology & Services Co., Ltd. (Shanghai, China) and are presented in Table 1.

**Table 1:** Primer sequences used in RT-qPCR

Targets	Forward (5′-3′)	Reverse (5′-3′)
miR-451a	ACACTCCAGCTGGGAAACCGTTACCATTAC	CTCAACTGGTGTCGTGGAGTCGGCAATTCAGTTGAGCTTACAG
DNMT3B	TAACAACGGCAAAGACCGAGGG	TCCTGCCACAAGACAAACAGCC
EPHA2	ACTGCCAGTGTCAGCATCAACC	GTGACCTCGTACTTCCACACTC
U6	AAAGCAAATCATCGGACGACC	GTACAACACATTGTTTCCTC
GAPDH	CTGGGCTACACTGAGCACC	AAGTGGTCGTTGAGGGCAATG

*Note*: *RT-qPCR* quantitative reverse transcriptase-PCR, *miR* microRNA, *DNMT3B* DNA methyltransferase 3B, *EPHA2* erythropoietin-producing hepatocellular receptor tyrosine kinase class A2, *GAPDH* glyceraldehyde-3-phosphate dehydrogenase

### Bioinformatics analysis

First, we downloaded the BCa-related differentially expressed miRNA dataset GSE113486 from the GEO database (https://www.ncbi.nlm.nih.gov/geo/), and conducted the differential analysis with the R Limma package. A heatmap was plotted using the R pheatmap package with Log FoldChange > 2, *p* value < 0.05 as the threshold. We then analyzed DNMT3B expression in BCa patients and normal bladder tissues through the oncomine database (https://www.oncomine.org/) and the GEPIA database (http://gepia.cancer-pku.cn/index.html). The relationship between miR-451a expression and the survival of BCa patients was analyzed using KM-plotter website (http://kmplot.com/analysis/). Moreover, targeting mRNAs of miR-451a were predicted through StarBase website (http://starbase.sysu.edu.cn/). Then the online bioinformatics tool DAVID 6.8 (https://david.ncifcrf.gov/) was used to carry out KEGG analysis of the selected 448 mRNAs and to map the bubble chart using the ggplot2.

### Cell culture and treatment

The T24 and 5637 cells were maintained in DMEM containing 10% FBS under 5% CO_2_ at 37 °C. Cells were collected for subsequent assays when the cell confluence reached 75–85%. T24 and 5637 cells in good growth condition were transfected with DNMT3B shRNA, sh-NC, mR-451a inhibitor, inhibitor NC, miR-451a mimic, mimic NC (Mock), oe-NC and oe-EPHA2 vectors alone or in combination. Transfection was performed using Lipofectamine 2000 kits according to the instructions. The cells were harvested at 48 h post-transfection, and RT-qPCR was used to verify the transfection efficiency of the cells.

### EdU assay

T24 and 5637 cells were seeded at 5000 cells/well in 96-well plates containing DMEM/F-12 medium and 50 mM EdU. After 12 h of culture, the cells were fixed with 4% paraformaldehyde, neutralized with 2 mg/mL glycine and infiltrated in 0.5% Triton X-100 for 10 min (all at room temperature). Apollo staining reaction buffer was then added for EdU immunostaining. The nuclei were labeled with Hoechst 33342, and EdU positive cells were counted from at least 500 cells under a fluorescence microscope (Olympus Ckx53, Olympus Optical Co., Ltd., Tokyo, Japan) and analyzed by Image J (National Institutes of Health, Bethesda, MD, USA).

### Flow cytometry

T24 and 5637 cells under good growth condition were detached with 0.25% trypsin. Totally 10^6^ cells were resuspended with Annexin V binding buffer (BioLegend, San Diego, CA, USA) and added with 5 μL APC Annexin V and 10 μL propidium iodide (PI) solution. The cells were loaded onto a C6 instrument (BD Biosciences, San Jose, CA, USA) after an incubation at room temperature in void of light for 15 min.

### TUNEL staining

The apoptosis was detected using TUNEL apoptosis detection kit (Promega) for T24 and 5637 cells. The cells were cultured with 20 μg/mL protease K at room temperature for 20 min and incubated with fluorescein isothiocyanate-TUNEL buffer in the dark for 1 h. Phosphate buffered saline without TdT enzymes was set as a negative control. The nuclei were labeled with Hoechst 33342, and the images were obtained under a microscope. The percentage of TUNEL positive cells was counted from at least 500 cells.

### Transwell assay

Cell invasion was detected using Transwell chambers (Corning Corporation, Midland, MI, USA). Briefly, the cells were placed into the apical chamber pre-coated with Matrigel. The basolateral chamber was filled with DMEM. After 48 h of incubation, cells in the basolateral chamber were fixed in 4% paraformaldehyde for 15 min and stained with 0.1% crystal violet for 20 min. Finally, cells in five randomly selected fields were counted under an inverted microscope (200×, Ckx53, Olympus Optical Co., Ltd., Tokyo, Japan). For migration assay, the operation procedure was consistent with the invasion assay except for Matrigel coating.

### RT-qPCR

Total RNA of tissues and cells was extracted with TRIzol (Sigma) reagent according to the protocols. miScript II RT kits (Takara Biotechnology Co., Ltd., Dalian, Liaoning, China) and SYBR Premix Ex TaqTM II (Takara) were used for reverse transcription and qPCR on an on-time ABI PRISM 7500 system.

### Western blot

Radio immunoprecipitation assay protein extraction reagents (Beyotime, Shanghai, China) containing phenylmethanesulfonyl fluoride (Roche, Basel, Switzerland) were used for lysis. The protein samples were then separated using SDS-PAGE and transferred to polyvinylidene fluoride membranes (Millipore, Billerica, MA, USA). The membranes were then sealed with 5% non-fat milk for 2 h and probed for one night at 4 °C. Subsequently, the membranes were re-probed at 37 °C with HRP-labeled secondary antibody for a period of 1 h. Chemiluminescence was detected by the ECL substrate kit (Thermo Fisher Scientific Inc., Waltham, MA, USA) and subsequent exposure using ChemiDoc XRS imaging systems. Band density was analyzed using Image J software (National Institutes of Health, Bethesda, MD, USA) and normalized to GAPDH.

### Promoter methylation detection

The genomic (g) DNA was isolated from T24 and 5637 cells with the help of the TIANamp gDNA kit (TIANGEN Biotech Co., Ltd., Beijing, China). Subsequently, the gDNA was converted into DNA sodium hydrogen sulfite using the EpiTect Plus Bisulfite kit (Qiagen company, Hilden, Germany).

The methylation status of miR-451a was determined by methylation-specific PCR (MSP) using a Veriti96PCR thermal cycler (Applied Biosystems, Inc., Foster City, CA, USA) with Taq PCR MasterMix (TIANGEN Biotech Co., Ltd., Beijing, China). PCR products were run on 3% agarose gel and subsequently identified by ethidium bromide staining at room temperature. 160 bp products represent miR-451a methylation and 120 bp products represent miR-451a demethylation.

### Chromatin immunoprecipitation (ChIP)

SimpleChIP Plus Sonication Chromatin IP (#56383S, Cell Signaling Technology, Danvers, MA, USA) was used for ChIP detection. About 1 × 10^7^ cells were crosslinked in 1% formaldehyde for 10 min and quenched in glycine for 5 min at room temperature. The dissolved chromatin was incubated with Flag and IgG antibodies.

### Luciferase activity assay

The wild-type (WT) EPHA2 promoter sequence containing binding sites with miR-451a was cloned into pmirGLO luciferase reporter vector (Promega), and mutant (MT) EPHA2 promoter sequence without binding sites was constructed into the same vector to generate report plasmids EPHA2-WT/EPHA2-MT. The 293 cells were co-transfected with 50 nM miR-451a mimic or NC mimic and 500 ng/mL reporter plasmids for 2 days. The luciferase intensity was assessed by a Dual-Glo® luciferase detection system (Promega).

### Xenografts

Four-week-old NOD/SCID mice (*n* = 20) were purchased from Shanghai SLAC Laboratory Animal Co., Ltd. (Shanghai, China). Nude mice were arbitrarily categorized into four groups, with five mice in each group. All mice were allowed to acclimatize for 1 week in the animal facility before treatment. T24 cells (2 × 10^6^) transfected with sh-NC, shDNMT3B, shDNMT3B + inhibitor NC or shDNMT3B + miR-451a inhibitor in 0.2 mL were injected subcutaneously in each flank of nude mice. Tumors were measured with a caliper at an interval of 5 days, and the tumor volume was calculated as π × width^2^ × length/6. The mice were euthanized by an intraperitoneal injection of excessive pentobarbital sodium after 35 days. At half an hour post-injection, the death of mice was confirmed by observing the lack of heartbeat, respiratory arrest, pupil dilation, and lack of nerve reflex. The tumor was subsequently collected for weight measurement and further examination. All animal experiments were compliant with ethic regulations and approved by Institutional Animal Care & Use Committee of the First Affiliated Hospital of Bengbu Medical College.

### Immunohistochemistry

First, the paraffin-embedded sections fixed in formalin were dewaxed with xylene and incubated with 3% hydrogen peroxide to block endogenous peroxidase activity. After that, the sections were treated with 5% goat serum and incubated overnight with the primary antibody at 4 °C. The sections were then incubated with the biotinylated anti-mouse/rabbit IgG and with streptomycin-biotin peroxidase for 15 min. The immunostained images were captured from 10 different fields using a fluorescence microscope (Olympus Ckx53) at a 400× magnification and analyzed by Image J.

### Statistical method

All experiments were repeated for at least three times. All data, presented by mean ± standard deviation (SD), were analyzed using the statistical software SPSS 22.0 (IBM, Chicago, IL, USA) and Prism 8.0 (GraphPad, San Diego, CA, USA). Data among multiple groups were tested by one-way or two-way analysis of variance (ANOVA) with Tukey’s post hoc test, and statistical significance was defined as a two-tailed value of *p* < 0.05.

## Results

### miR-451a is downregulated in serum of BCa patients and its promoter is methylated

**Fig. 1:**
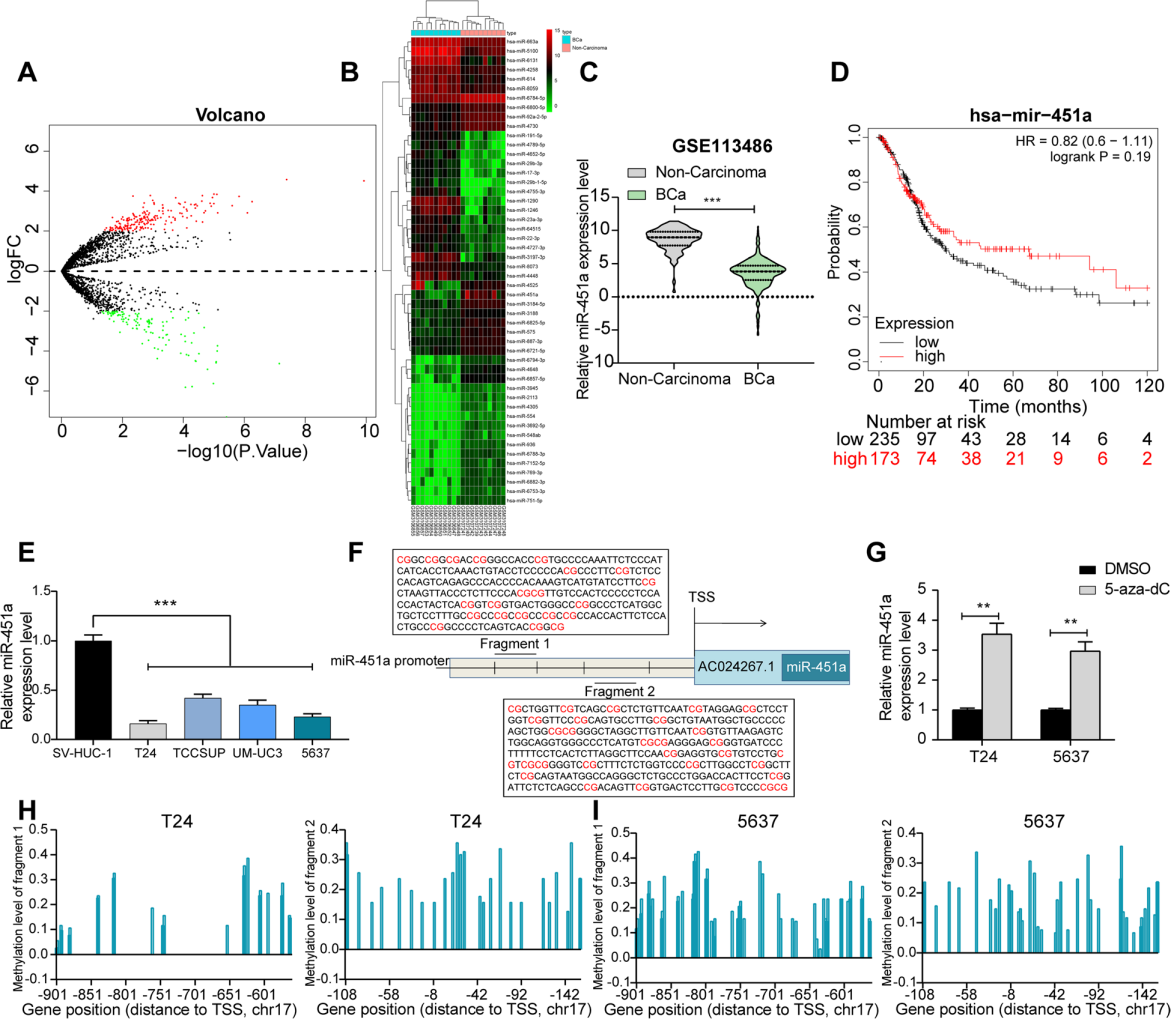
miR-451a expresses poorly in serum of patients with BCa and its promoter is methylated in BCa cells. **a**, the volcanic map of 114 upregulated miRNAs and 195 downregulated miRNAs in serum of 12 BCa patients and 10 healthy controls in GSE113486 dataset; **b**, the heatmap of the top 50 differentially expressed miRNAs; **c**, serum expression of miR-451a in 392 BCa patients and 100 healthy controls in GSE113486 dataset; **d**, the relationship between miR-451a and survival of patients predicted by the KM-plotter website; **e**, miR-451a expression in BCa cell lines T24, 5637, TCCSUP, UM-UC3 and SV-HUC-1 cells determined by RT-qPCR; **f**, miR-451a chromosomal mapping and CpG island prediction; **g**, miR-451a expression in T24 and 5637 cells delivered with 5-aza-dC; **h**, promoter methylation in T24 cells (the top 3 haplotypes of high frequency were presented); **i**, promoter methylation in 5637 cells (the top 3 haplotypes of high frequency were presented). Data are represented as the means ± SD from three independent assays. Statistically significant differences were calculated using unpaired *t*-test (panel **c**), one-way (panel **e**) or two-way ANOVA (panel **g**), followed by Tukey’s multiple comparison test. **p < 0.01 vs DMSO treatment, ***p < 0.001 vs SV-HUC-1 cells or non-carcinoma

Firstly, 309 differentially expressed miRNAs from the serum of 12 BCa patients and 10 healthy controls were screened out in GSE113486 by setting Log FoldChange > 2, *p* value < 0.05 as the screening thresholds. Among them, 114 miRNAs were upregulated and 195 miRNAs were downregulated in BCa (Fig. 1a). The heatmap in Fig. 1b shows the top 50 differentially expressed miRNAs. Subsequently, we found that miR-451a was significantly reduced (Fig. 1c) in the serum of 392 BCa patients relative to that in 100 healthy control patients in the GSE113486 dataset. Moreover, the KM-plotter website revealed that BCa patients highly expressing miR-451a had longer survival time (Fig. 1d). We then examined the expression pattern of miR-451a in BCa cell lines T24, 5637, TCCSUP and UM-UC3 and immortalized human uroepithelial cells SV-HUC-1. The expression of miR-451a in BCa cell lines was significantly inhibited (Fig. 1e). These results suggest that miR-451a may play a potential regulatory role in BCa.

miR-451a is located on the chr17 chromosome and belongs to the AC024267.1 region (Fig. 1f). It has been previously displayed that miR-433 was frequently downregulated in BCa due to the methylation status of CpG islands in the promoter region [11]. To assess the AC024267.1 promoter methylation status and the regulatory role of miR-451a in BCa, miR-451a expression was increased in T24 and 5637 cell lines after 5′-aza-2′-deoxycytidine (5-aza-dC) treatment, as revealed by RT-qPCR (Fig. 1g). Moreover, promoter methylation detection was performed to test the methylation level of miR-451a CpG island in T24 and 5637 cell lines. The results suggested that promoter methylation was responsible for miR-451a dysregulation in BCa (Fig. 1h, i). Therefore, the results indicated that miR-451a was downregulated in BCa due to hypermethylation of CpG islands, whereas miR-451a may play a tumor suppressor role in the BCa.

### DNMT3B promotes miR-451a promoter methylation

**Fig. 2:**
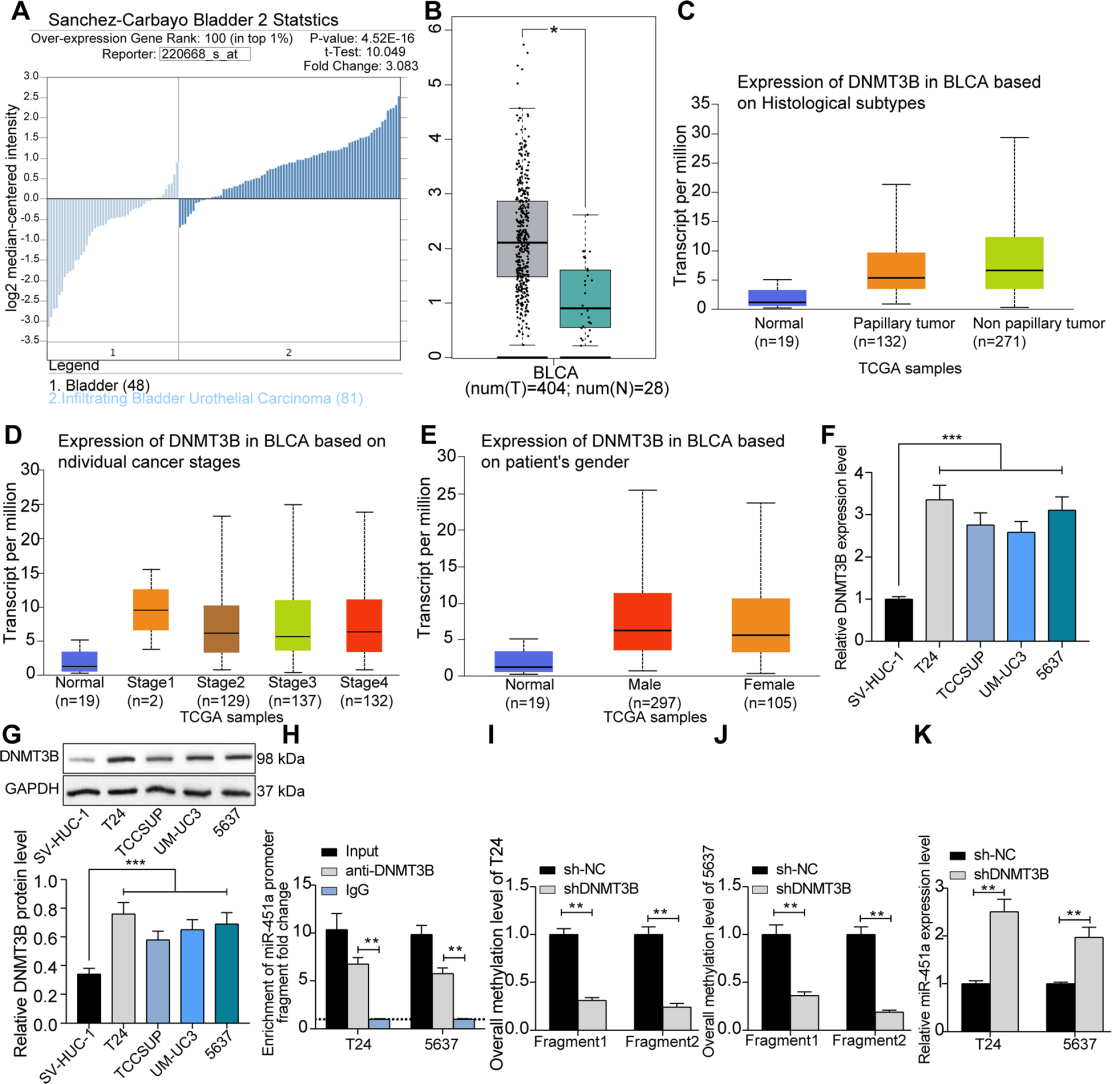
DNMT3B promotes miR-451a promoter methylation. **a**, DNMT3B expression in BCa and matched adjacent tissues in the Oncomine database; **b**, DNMT3B expression in normal and tumor tissues in TCGA databases predicted by the GEPIA website; **c**, the association between DNMT3B expression and the histological subtype of BC patients; **d**, the association between DNMT3B expression and the clinical stage of BC patients; **e**, the association between DNMT3B expression and the gender of BCa patients; **f**, DNMT3B mRNA expression in BCa cell lines T24, 5637, TCCSUP, UM-UC3 and SV-HUC-1 cells determined by RT-qPCR; **g**, DNMT3B protein expression in BCa cell lines T24, 5637, TCCSUP, UM-UC3 and SV-HUC-1 cells determined by western blot (full-length blots/gels are presented in Supplementary Figure 1A, B); **h**, the binding relationship between DNMT3B and miR-451a promoter in T24 and 5637 cells tested by ChIP-qPCR; **i**, promoter methylation in T24 cells in response to shDNMT3B (the top 3 haplotypes of high frequency were presented); **j**, promoter methylation in 5637 cells in response to shDNMT3B (the top 3 haplotypes of high frequency were presented); **k**, miR-451a expression in BCa cell lines in response to shDNMT3B determined by RT-qPCR. Data are represented as the means ± SD from three independent assays. Statistically significant differences were calculated using unpaired *t*-test (panel **b**), one-way (panel **c** and **d**) or two-way ANOVA (panel **f-k**), followed by Tukey’s multiple comparison test. *p < 0.05 vs tumor tissues, **p < 0.01 vs sh-NC or IgG treatment, ***p < 0.001 vs SV-HUC-1 cells

To further investigate the upstream regulatory mechanism of miR-451a, we found through literature review that DNMTs could inhibit miR-200a expression by promoting methylation of the miR-200a promoter, thus promoting breast cancer cell growth [12]. Therefore, we first found that DNMT3B was significantly overexpressed (Fig. 2a) in the BCa through the Oncomine database, and a same trend was observed in the bladder urothelial carcinoma (BLCA) tissues using the GEPIA website (Fig. 2b). The association between the expression of DNMT3B and the clinical stage, histological subtype, and gender of the patients in The Cancer Genome Atlas (TCGA) database was queried. We found no significant difference between the expression of DNMT3B and the histological subtype (Fig. 2c), clinical stage (Fig. 2d) or gender (Fig. 2e) of BCa patients. Subsequently, a significant increase was observed in DNMT3B expression in BCa cell lines compared to SV-HUC-1 cells (Fig. 2f, g). Then, we used ChIP-qPCR experiments to find that fragments of miR-451a promoters in chromatin complexes precipitated using specific antibody against DNMT3B were significantly higher than IgG (Fig. 2h). Moreover, the methylation level of miR-451a promoters in T24 and 5637 cells was reduced (Fig. 2i, j) after DNMT3B knockdown, and miR-451a expression in cells was significantly increased (Fig. 2k).

### Overexpression of miR-451a or knockdown of DNMT3B inhibits the aggressiveness of BCa cells in vitro

**Fig. 3:**
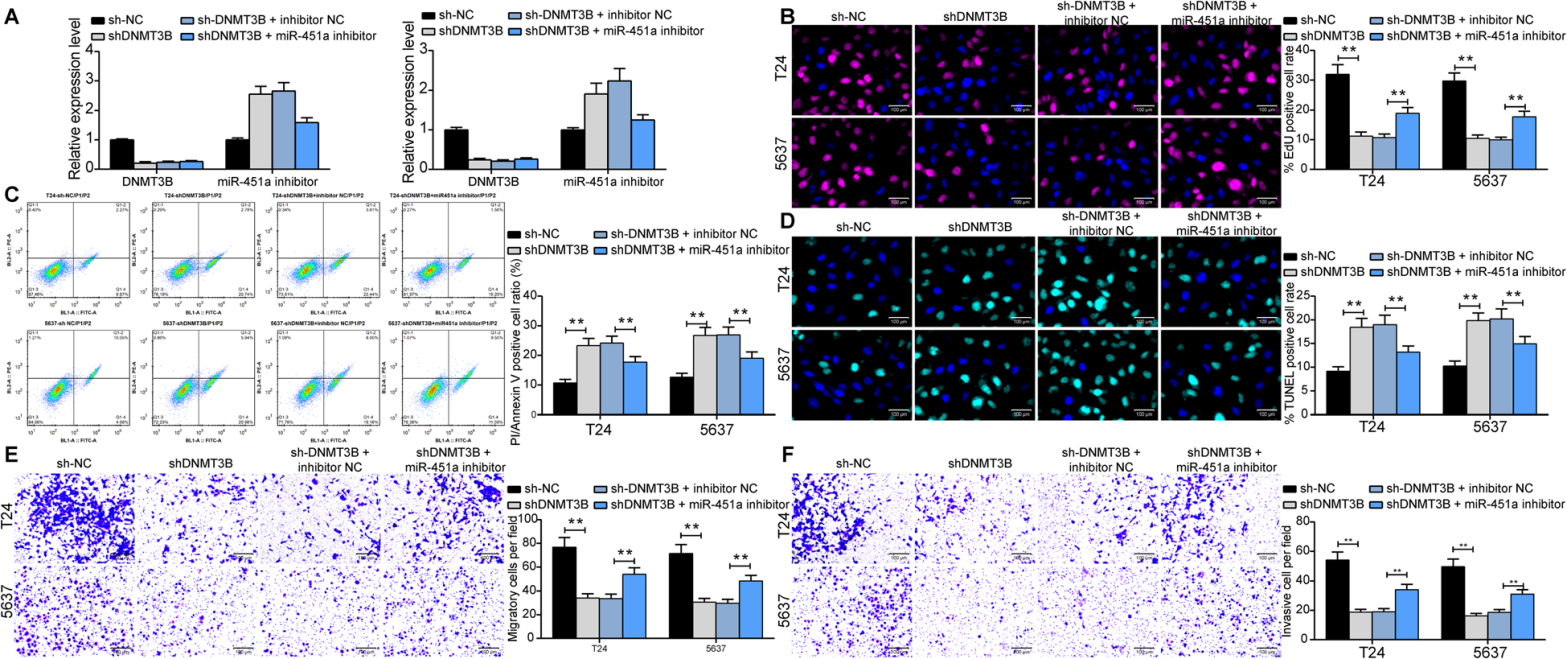
Overexpression of miR-451a or knockdown of DNMT3B inhibits the aggressiveness of BCa cells in vitro. BCa cells were transfected with shDNMT3B alone or with miR-451a inhibitor. **a**, miR-451a expression in BCa cell lines determined by RT-qPCR; **b**, the number of T24 and 5637 cells at the S phase determined by EdU staining where red represents the replicating DNA stained by EdU and blue represents the nucleus stained by DAPI; **c**, apoptosis in T24 and 5637 cells detected by flow cytometric analysis; **d**, the formation of apoptotic bodies in T24 cells and 5637 cells determined by TUNEL staining where green represents apoptotic bodies stained by TUNEL and blue represents nuclei stained by DAPI; **e**, number of migratory T24 and 5637 cells was detected by Transwell assays; **f**, number of invasive T24 and 5637 cells was detected by Transwell assays. Data are represented as the means ± SD from three independent assays. Statistically significant differences were calculated using one-way or two-way ANOVA, followed by Tukey’s multiple comparison test. **p < 0.01 vs sh-NC or sh-DNMT3B + inhibitor NC treatment

In order to assess the role of miR-451a and DNMT3B in the aggressiveness of BCa cells, we first knocked-down DNMT3B expression in T24 and 5637 cells. The results of the Fig. 2h demonstrated that miR-451a in these cells increased significantly after knocking down DNMT3B. Therefore, we further knocked-down miR-451a in cells, and RT-qPCR verified the successful transfection (Fig. 3a). The cell proliferation activity was repressed notably by shDNMT3B, which was restored by miR-451a inhibitor (Fig. 3b). Then, after PI/Annexin V staining and flow cytometry, the number of apoptotic T24 and 5637 cells was elevated significantly under the action of shDNMT3B, and further addition of miR-451a inhibitor weakened the promoting role of shDNMT3B in the apoptosis of BCa cells (Fig. 3c). The TUNEL assay also exhibited the same experimental results (Fig. 3d). Then, we further used Transwell assays to detect the invasion and migration ability of cells. The number of cells that migrated or invaded to the basolateral chamber was significantly reduced after DNMT3B knockdown, but miR-451a inhibitor conferred migratory and invasive potentials to T24 and 5637 cells (Fig. 3e, f).

### miR-451a impairs the PI3K/Akt signaling activity by targeting EPHA2

**Fig. 4:**
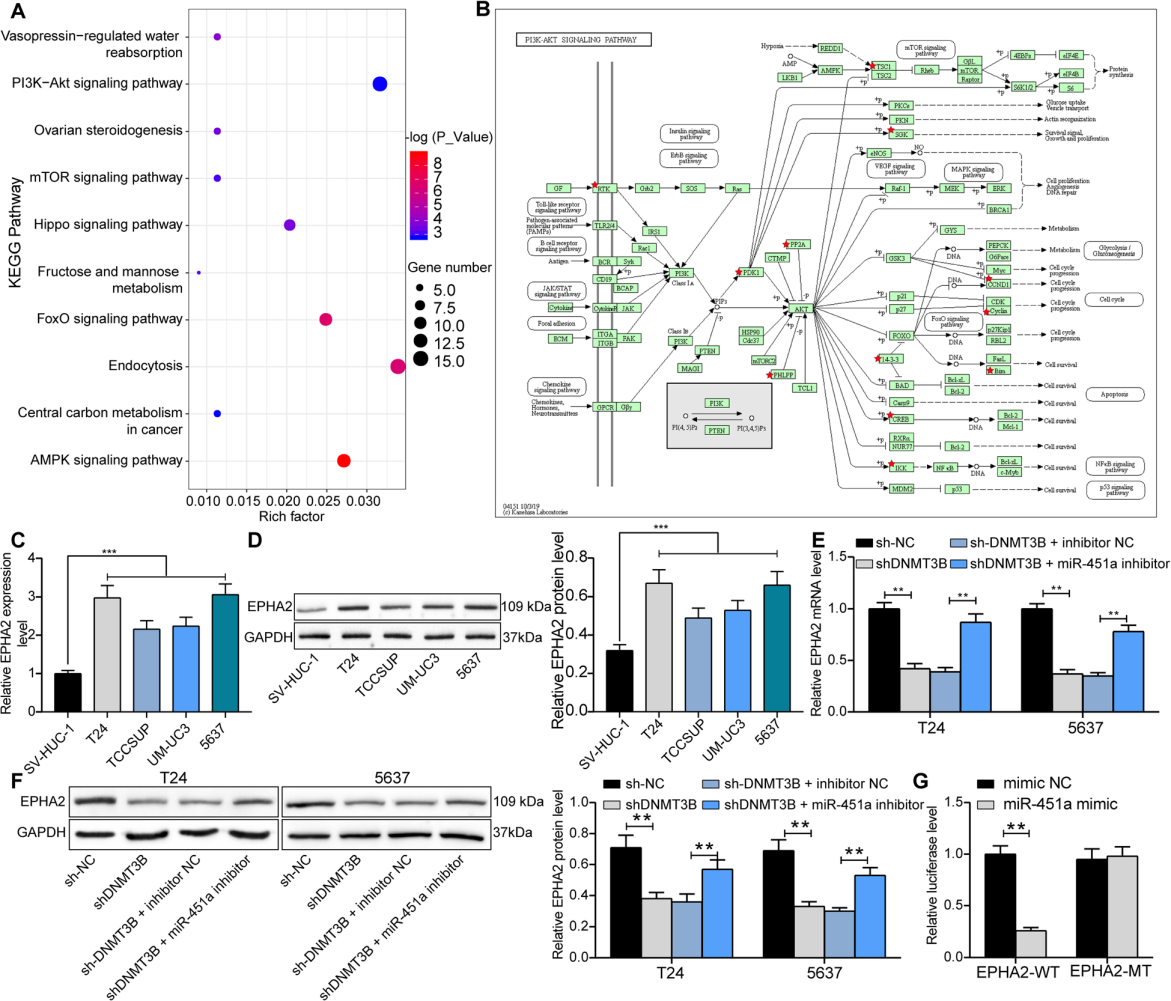
miR-451a targets EPHA2 to regulate the PI3K/AKT signaling. **a**, the targeting mRNAs of miR-451a predicted by StarBase and analyzed using a DAVID 6.8 online bioinformatics analysis tool. Bubble plots showed 14 signaling pathways; **b**, gene distribution in the PI3K/AKT signaling in the KEGG database; **c**, EPHA2 mRNA expression in BCa cell lines T24, 5637, TCCSUP, UM-UC3 and SV-HUC-1 cells determined by RT-qPCR; **d**, EPHA2 protein expression in BCa cell lines T24, 5637, TCCSUP, UM-UC3 and SV-HUC-1 cells determined by western blot (full-length blots/gels are presented in Supplementary Figure 2A, B); **e**, EPHA2 mRNA expression in BCa cell lines after transfection determined by RT-qPCR; **f**, EPHA2 protein expression in BCa cell lines after transfection determined by western blot (full-length blots/gels are presented in Supplementary Figure 3A-D); **g**, the binding relationship between miR-451a and EPHA2 verified by dual-luciferase assays. Statistically significant differences were calculated using one-way or two-way ANOVA, followed by Tukey’s multiple comparison test. **p < 0.01 vs sh-NC or sh-DNMT3B + inhibitor NC treatment, ***p < 0.001 vs SV-HUC-1 cells

We focused our attention on miR-451a downstream regulatory mechanisms after studying the upstream regulatory mechanisms of miR-451a. The targeting mRNAs of miR-451a were firstly predicted by StarBase bioinformatics prediction website. A total of 458 mRNAs were screened out. After that, we used DAVID bioinformatics online tool for enrichment analysis, and 14 signaling pathways were enriched (Table 2). The PI3K/AKT signaling was significantly enriched (Fig. 4a), and the activation the PI3K/AKT signaling was reported to significantly promote BCa cell proliferation and migration [13, 14]. Moreover, we found EPHA2 was in the upstream (Fig. 4b) of the PI3K/AKT signaling, and the tumor initiating role of EPHA2 has been indicated in BCa [15]. We subsequently examined the expression of EPHA2 in T24, 5637, TCCSUP, UM-UC3 and SV-HUC-1 cells and noted that its expression in BCa cells were much higher than those in SV-HUC-1 cells (Fig. 4c, d). In addition, after DNMT3B knockdown, EPH2A expression in T24 and 5637 cells was significantly inhibited, while EPHA2 expression was recovered (Fig. 4e, f) under the synergistic effect of shDNMT3B and miR-451a inhibitor. Consequently, we used dual-luciferase assays to test the binding relationship between miR-451a and EPHA2 3’UTR. The luciferase activity was significantly inhibited in 293 T cells delivered with miR-451a and EPHA2-WT (Fig. 4g).

**Table 2:** Enrichment analysis

Term	Count	*p* value
hsa04152AMPK signaling pathway	12	0
hsa04068FoxO signaling pathway	11	0.001
hsa04144Endocytosis	15	0.002
hsa04962Vasopressin-regulated water reabsorption	5	0.019
hsa04390Hippo signaling pathway	9	0.026
hsa04913Ovarian steroidogenesis	5	0.028
hsa00051Fructose and mannose metabolism	4	0.039
hsa04150mTOR signaling pathway	5	0.047
hsa04151PI3K-Akt signaling pathway	14	0.042
hsa05230Central carbon metabolism in cancer	5	0.041
hsa04022cGMP-PKG signaling pathway	8	0.009
hsa00100Steroid biosynthesis	3	0.014
hsa04622RIG-I-like receptor signaling pathway	5	0.002
hsa05205Proteoglycans in cancer	9	0.019

### The PI3K/AKT signaling is disrupted by shDNMT3B

**Fig. 5:**
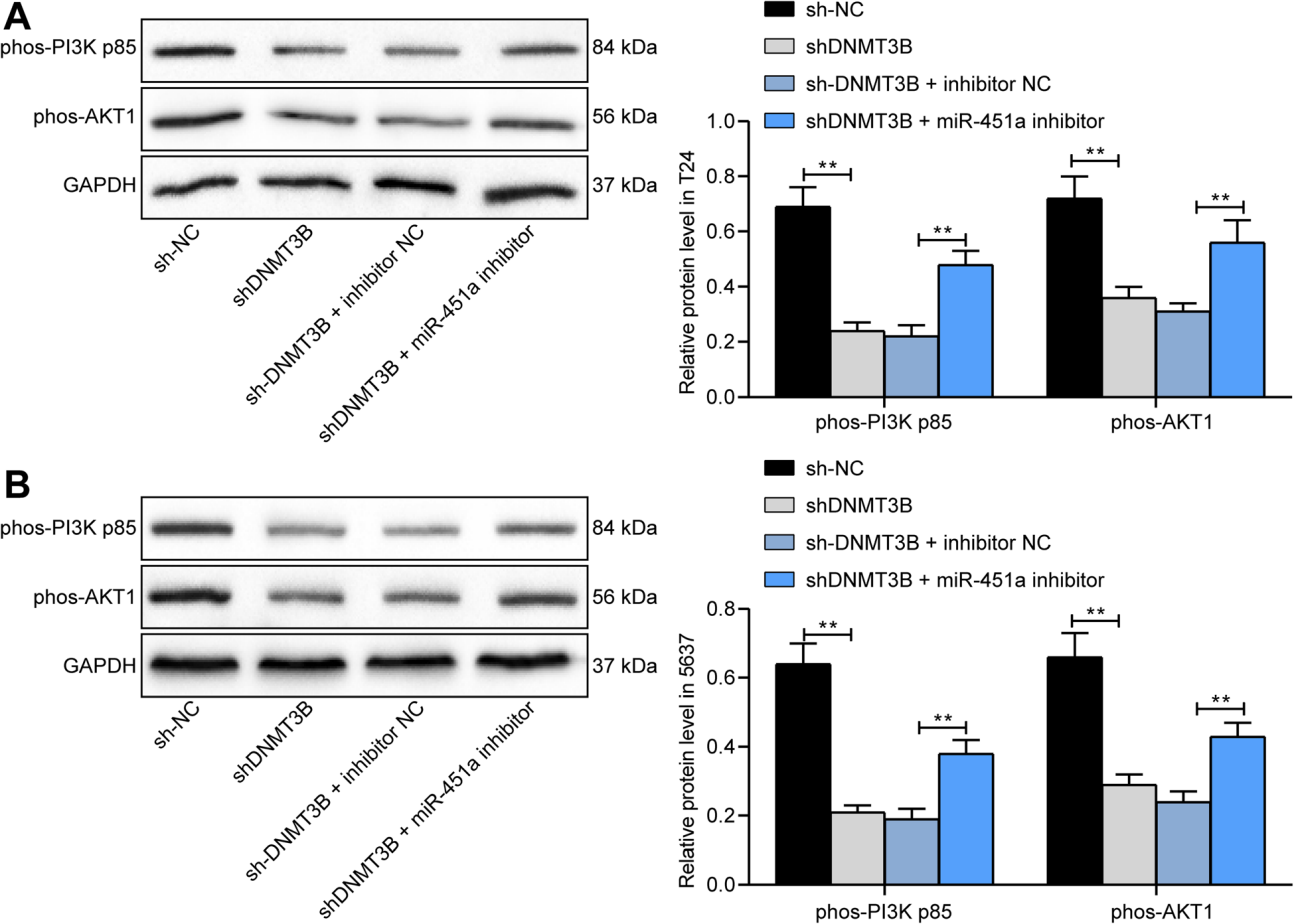
The PI3K/AKT signaling is blocked by DNMT3B knockdown. T24 and 5637 cells were treated with shDNMT3B alone or in the presence of miR-451a inhibitor. **a**, the extents of PI3K and AKT phosphorylation in T24 cells determined by western blot (full-length blots/gels are presented in Supplementary Figure 4A-C); **b**, the extent of PI3K and AKT phosphorylation in 5637 cells determined by western blot (full-length blots/gels are presented in Supplementary Figure 5A-C). Statistically significant differences were calculated using one-way or two-way ANOVA, followed by Tukey’s multiple comparison test. **p < 0.01 vs sh-NC or sh-DNMT3B + inhibitor NC treatment

On the basis of our findings in Fig. 4, we observed that the PI3K/AKT signaling was significantly enriched, so we used western blot to assess the extent of PI3K p85 and AKT1 phosphorylation in T24 and 5637 cells. We found that their phosphorylation levels were significantly inhibited in cells poorly-expressed DNMT3B. The extents of PI3K p85 and AKT1 phosphorylation were significantly restored (Fig. 5a, b) after further transfection using miR-451a inhibitor.

### miR-451a-mediated EPHA2 silencing inhibits malignant biological behavior of BCa cells

**Fig. 6:**
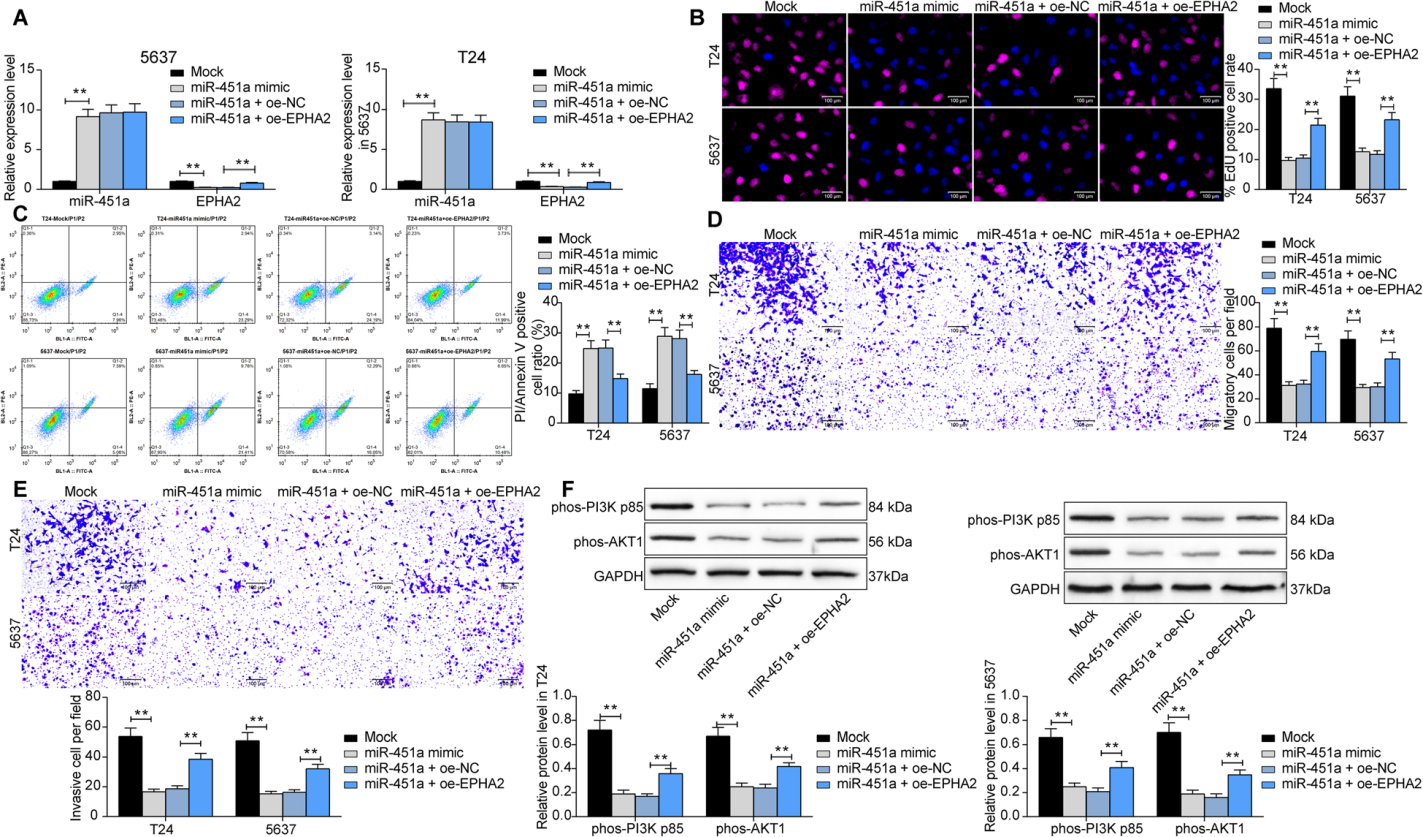
miR-451a-mediated EPHA2 silencing inhibits malignant biological behavior of BCa cells. T24 and 5637 cells were transfected with miR-451a mimic alone or in the presence of oe-EPHA2. **a**, miR-451a expression and the mRNA expression of EPHA2 in BCa cells after transfection assessed by RT-qPCR; **b**, the number of BCa cells in the S phase determined by EdU staining where red represents the replicating DNA stained by EdU and blue represents the nucleus stained by DAPI; **c**, apoptosis in T24 and 5637 cells detected by flow cytometry; **d**, number of migratory BCa cells assessed by Transwell assays; **e**, number of invasive T24 and 5637 cells was detected by Transwell assays; **f**, the extents of PI3K and AKT phosphorylation in T24 cells (full-length blots/gels are presented in Supplementary Figure 6A-C) and 5637 cells (full-length blots/gels are presented in Supplementary Figure 7A-C) determined by western blot. Data are represented as the means ± SD from three independent assays. Statistically significant differences were calculated using one-way or two-way ANOVA, followed by Tukey’s multiple comparison test. **p < 0.01 vs Mock or miR-451a + oe-NC treatment

To further validate the effect of the miR-451a/EPHA2 axis on BCa cell growth, we overexpressed miR-451a in T24 and 5637 cells, and further transfected EPHA2 overexpression plasmids in cells. We validated the transfection was successful (Fig. 6a) by RT-qPCR. miR-451a mimic notably inhibited the proliferative activity of T24 and 5637 cells; however, further increasing the expression of EPHA2 in the cells remarkably weakened the inhibition of miR-451a mimic on cell activity (Fig. 6b). Moreover, we further analyzed T24 and 5637 cell apoptosis by flow cytometry after PI/Annexin V staining. Overexpression of miR-451a significantly promoted cell apoptosis, but oe-EPHA2 significantly inhibited miR-451a mimic-induced apoptosis (Fig. 6c). Subsequently, we used Transwell assays to verify the migration and invasion capacities of T24 and 5637 cells. The cell migration and invasion were significantly hampered after overexpression of miR-451a, which was mitigated by oe-EPHA2 (Fig. 6d, e). Lastly, the extent of PI3K/AKT phosphorylation in T24 and 5637 cells was assessed. Similarly, the blockage of miR-451a mimic on the PI3K/AKT signaling was antagonized by oe-EPHA2 (Fig. 6f).

### The DNMT3B/miR-451a/EPHA2/PI3K/AKT axis involves in the progression of BCa in vivo

**Fig. 7:**
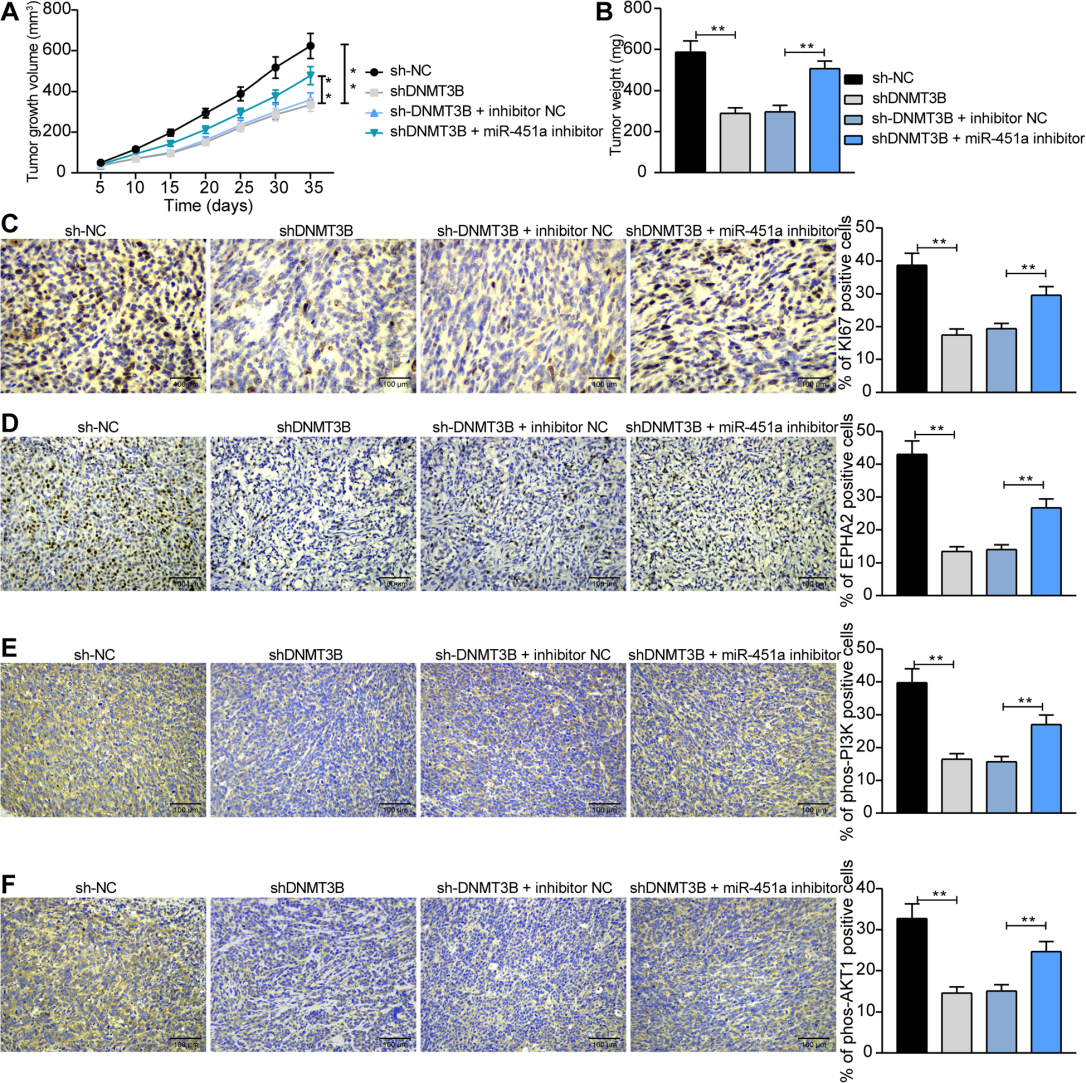
DNMT3B/miR-451a/EPHA2/PI3K/AKT axis modulates BCa cell growth in vivo. T24 cells (2 × 10^6^ cells/mL) with stable poor expression of DNMT3B or miR-451a were injected into the right armpit of NOD/SCID mice. **a**, from the fifth day, the growth rate of T24 cells was evaluated by measuring the volume every 5 days in vivo; **b**, tumor weight; **c**, KI67 staining intensity in tumors evaluated by immunohistochemistry; **d**, EPHA2 staining intensity in tumors evaluated by immunohistochemistry; **e**, phos-PI3K staining intensity in tumors evaluated by immunohistochemistry; **f**, phos-AKT1 staining intensity in tumors evaluated by immunohistochemistry. Data are represented as the means ± SD from three independent assays. Statistically significant differences were calculated using one-way or two-way ANOVA, followed by Tukey’s multiple comparison test. **p < 0.01 vs mice injected with cells transfected with sh-NC or sh-DNMT3B + inhibitor NC

To assess the effect of the DNMT3B/miR-451a axis on the development of BCa, we injected T24 cells (2 × 10^6^ cells/mL) stably poor-expressing DNMT3B or miR-451a into the right armpit of NOD/SCID mice. Tumor volume was measured every 5 days from the fifth day after injection to assess the growth rate of T24 cells in vivo. The growth rate of T24 cells with poor expression of DNMT3B was significantly decreased, but under the further action of miR-451a inhibitor, the growth rate of T24 cells in vivo was significantly increased (Fig. 7a). On the 35th day after injection, the weight of tumors formed by T24 cells in NOD/SCID mice showed the same experimental trend (Fig. 7b) as the volume. We then used immunohistochemistry to detect the staining intensity of KI67, EPHA2, phos-PI3K and phos-AKT1 in tumors. The staining intensity of KI67, EPHA2, phos-PI3K and phos-AKT1 in tumors decreased significantly after DNMT3B silencing. While in the presence of miR-451a inhibitor, the staining intensity of KI67, EPHA2, phos-PI3K and phos-AKT1 was notably restored (Fig. 7c-f).

## Discussion

BCa is the 7th most prevalent cancer in male and the 17th most prevalent in female in a global range [16]. The miRNA signatures in BCa have been characterized, but the exact role of miRNA in the pathogenesis of BCa remains much less explored. In this study, the expression of miR-451a was significantly lower in serum of BCa patients than in normal controls, which was tightly linked to the hypermethylation of its promoter region. In addition, miR-451a promoter region was methylated by DNMT3B. Moreover, loss- and gain-of-function analyses manifested that DNMT3B knockdown curtailed the proliferation, migration, and invasion of BCa cells, while induced apoptosis, which were reversed by miR-451a inhibitor. Further investigation disclosed that the EPHA2/PI3K/AKT axis was participated in the BCa cell growth mediated by DNMT3B and miR-451a. These findings have highlighted the carcinostatic role of miR-451a in BCa.

The carcinostatic role of miR-451a has been substantiated in papillary thyroid carcinoma [17], lung cancer [18] as well as prostate cancer [19]. Likewise, our bioinformatics tools revealed that BCa patients with higher miR-451a expression benefitted from longer survival, hinting the possible prognostic role of miR-451a in BCa. In addition, we confirmed that the dysregulation of miR-451a in BCa was associated with hypermethylation of its promoter region. In hepatocellular carcinoma cells, the CpG islands surrounding miR-433 were methylated, and the DNA methylation agent 5-aza-dC notably stimulated the expression of miR-433 [20]. Consistently, 5-aza-dC also induced miR-451a expression in BCa cells in the current study, further confirming the association between miR-451a downregulation and hypermethylation in BCa cells. miR-29b-3p has been validated to regulate the progression of BCa via targeting DNMT3B [21]. Conversely, the expression of some miRNAs can be modulated by DNA methylation, which indicated the linkages between miRNAs and epigenetic mechanisms [22]. For instance, Flap endonuclease 1 was proved to interact with DNMT3A to repress miR-200a-5p expression mediated by methylation in breast cancer [12]. In line with those previous evidence, our assays validated that knockdown of DNMT3B reduced miR-451a methylation, while enhanced miR-451a expression in BCa. In addition, rescue experiments suggested that the inhibitory role of DNMT3B knockdown on BCa cell proliferation, migration and invasion was abrogated by miR-451a inhibitor, further evidencing the interaction between DNMT3B and miR-451a as well as their roles in BCa.

Subsequently, our KEGG enrichment suggested that the PI3K/AKT signaling was recruited in BCa. In fact, 40% of BCa patients demonstrated the PI3K/AKT signaling overactivation [23]. In addition, targeting this signaling has been reported as a possible therapeutic target in BCa [24, 25]. Our observations displayed that DNMT3B knockdown disrupted the PI3K/AKT signaling in BCa cells, which was reversed by miR-451a inhibitor. Multi-omics analysis and spontaneous metastatic mouse models performed by So et al. also documented that DNMT3B altered multiple pathways including PI3K/AKT [26]. While miR-451a played a tumor suppressive role in gastric cancer via mediation of the PI3K/AKT signaling [13]. Furthermore, EPHA2 was identified to be an upstream gene of the PI3K/AKT signaling by our KEGG analysis. EPHA2 was found to be required for progranulin-induced biological activity and cisplatin chemosensitivity in BCa [15]. Besides, EPHA2 was involved in the inhibition of angiogenesis mediated by leflunomide in BCa [27]. Promoted EPHA2 expression in cancer cells is linked to a dismal prognosis because of recurrence as a consequence of enhanced metastasis [28]. As for its upstream miRNAs, EPHA2 has been indicated to be targeted by different miRNAs to participate the development of various cancers, including non-small cell lung cancer [29], hepatocellular carcinoma [30] as well as colorectal cancer [31]. After the validation of the targeting relationship between EPHA2 and miR-451a in BCa cells, we carried out rescue experiments again. A series of assays validated that overexpression of EPHA2 rescued the tumor suppressive role of miR-451a in BCa cells. Our in vitro findings were reproduced on nude mice where shDNMT3B slowed down tumor growth, while miR-451a inhibitor partially restored the tumorigenicity of T24 cells with the involvement of EPHA2/PI3K/AKT axis.

## Conclusion

**Fig. 8:**
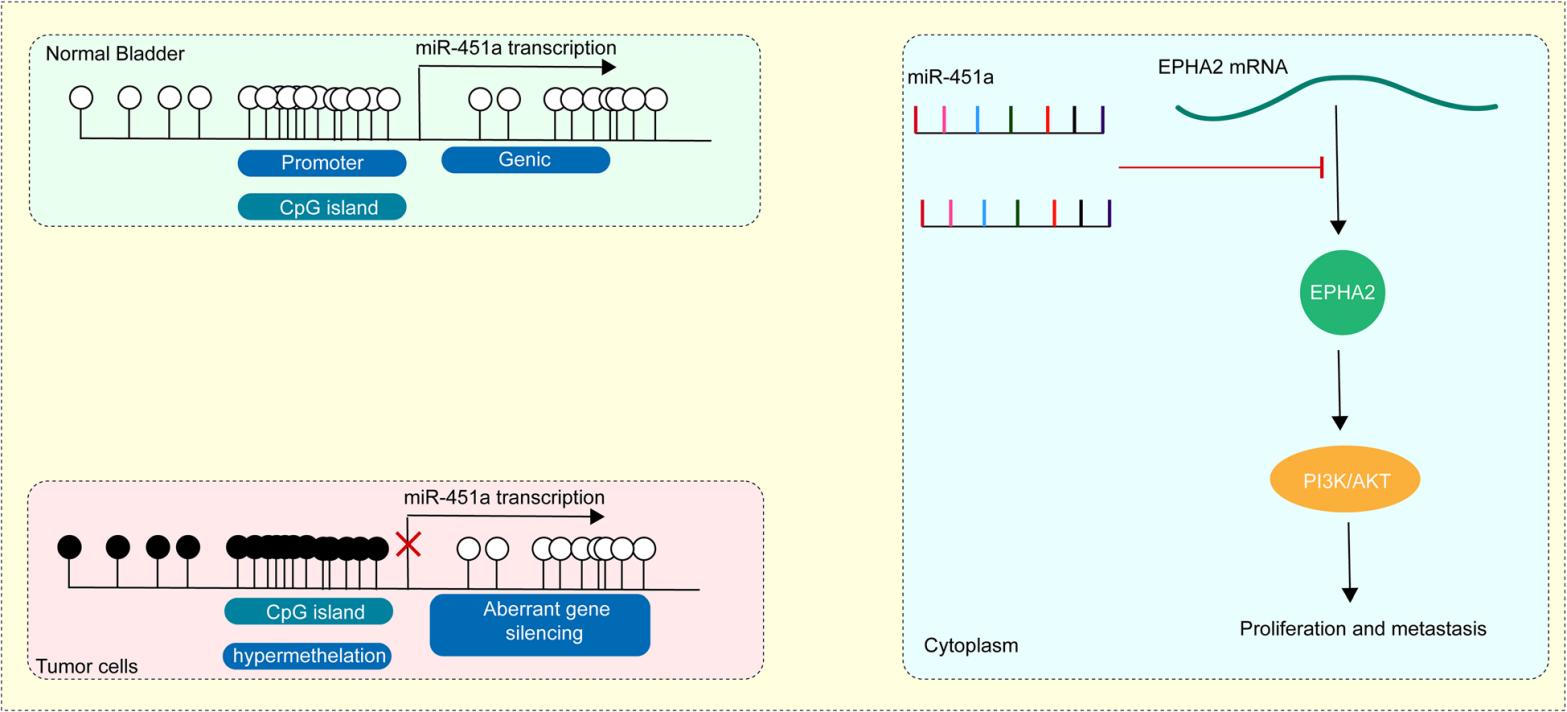
Schematic diagram. DNMT3B inhibits miR-451a transcription by methylation modification of the miR-451a promoter, thus activating EPHA2 and the following PI3K/AKT signaling, leading to BCa progression

Our study demonstrated that miR-451a was reduced in BCa cells. Poor expression of miR-451a was associated with its promoter methylation induced by DNMT3B. In addition, we proposed that miR-451a played an essential role in the proliferation, migration, and invasion of BCa cells by binding to EPHA2 and impairing the PI3K/AKT signaling (Fig. 8). Therefore, miR-451a may act as a prognostic marker for BCa patients and a potential target for the treatment of BCa.

## Supplementary information

**Additional file 1.**

## Data Availability

All the data generated or analyzed during this study are included in this published article.

## Abbreviations

ANOVA: Analysis of variance
BCa: Bladder cancer
BLCA: Bladder urothelial carcinoma
ChIP: Chromatin immunoprecipitation
DNMT3B: DNA methyltransferase 3B
EPHA2: Erythropoietin-producing hepatocellular receptor tyrosine kinase class A2
FBS: Fetal bovine serum
GAPDH: Glyceraldehyde-3-phosphate dehydrogenase
GEO: Gene Expression Omnibus
HRP: Horseradish peroxidase
KEGG: Kyoto Encyclopedia of Genes and Genomes
MSP: Methylation-specific PCR
PI: Propidium iodide
TUNEL: Terminal deoxynucleotidyl transferase (TdT)-mediated dUTPbiotin nick end labeling
DMEM: Dulbecco’s modified Eagle’s medium
