# Supplementary figures and images for: microRNA-451a promoter methylation regulated by DNMT3B expedites bladder cancer development via the EPHA2/PI3K/AKT axis
Source: BMC Cancer. 2020 Oct 21;20:1019. doi: 10.1186/s12885-020-07523-8 (PMC7579823; doi:10.1186/s12885-020-07523-8)

**Supplementary Figures**

**
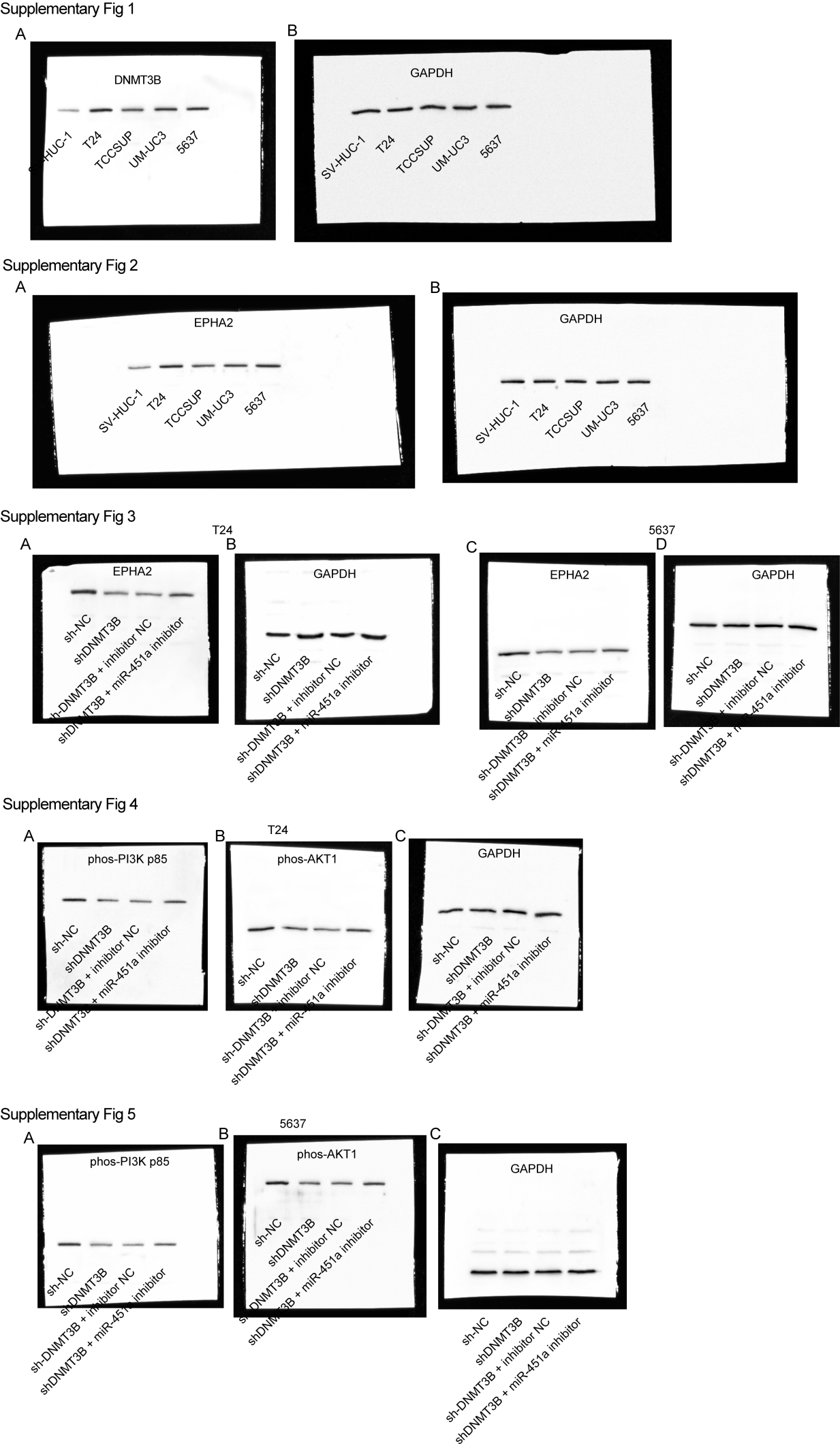
**

**
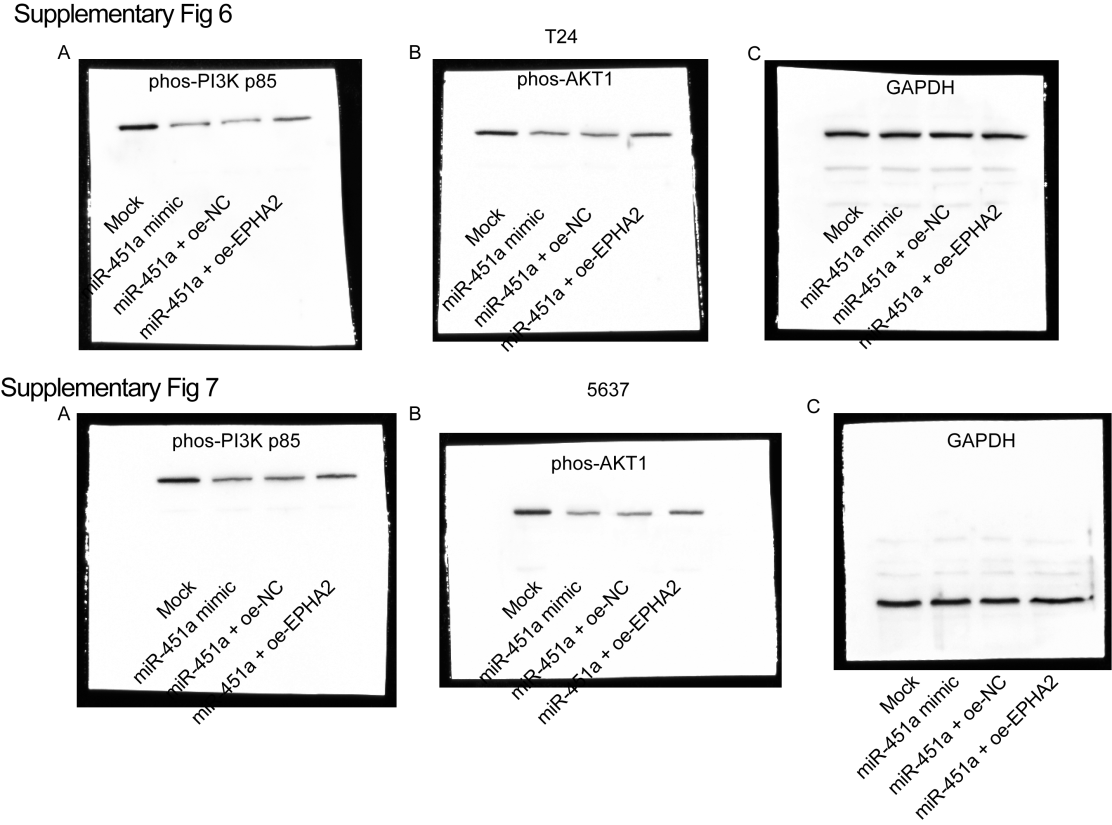
**

Supplement: Supplementary file 1 — Additional file 1. [file 12885_2020_7523_MOESM1_ESM.docx]
